# Challenges for bovine viral diarrhoea virus antibody detection in bulk milk by antibody enzyme-linked immunosorbent assays due to changes in milk production levels

**DOI:** 10.1186/s13028-015-0125-z

**Published:** 2015-06-23

**Authors:** Alessandro Foddai, Claes Enøe, Anders Stockmarr, Kaspar Krogh, Åse Uttenthal

**Affiliations:** Section of Epidemiology, National Veterinary Institute, Technical University of Denmark, Bülowsvej 27, DK-1870 Frederiksberg C, Denmark; Statistics and Data Analysis Section, Department of Applied Mathematics and Computer Science, Technical University of Denmark, Matematiktorvet, DK-2800 Lyngby, Denmark; Knowledge Centre for Agriculture, Cattle, Danish Cattle Federation, Agro Food Park 15, Skejby, 8200 Aarhus N, Denmark; Section for Virology, National Veterinary Institute, Technical University of Denmark, Lindholm, DK-4771 Kalvehave, Denmark

**Keywords:** Bovine viral diarrhoea, Bulk milk, Antibody ELISA, Surveillance

## Abstract

**Background:**

Bovine viral diarrhoea (BVD) is considered eradicated from Denmark. Currently, very few (if any) Danish cattle herds could be infected with BVD virus (BVDV). The Danish antibody blocking enzyme-linked immunosorbent assay (ELISA) has been successfully used during the Danish BVD eradication program, initiated in 1994. During the last decade, the cattle herd size has increased while the prevalence of BVDV has decreased. In this study, we investigated how these changes could affect the performance of the Danish blocking ELISA and of the SVANOVIR^®^BVDV-Ab indirect ELISA. The latter has successfully been used to eradicate BVD in Sweden.

Data (2003–2010) on changes in median herd size and milk production levels, occurrence of viremic animals and bulk milk surveillance were analysed. Additionally, the Danish blocking ELISA and the SVANOVIR ELISA were compared analyzing milk and serum samples. The prevalence of antibody positive milking cows that could be detected by each test was estimated, by diluting positive individual milk samples and making artificial milk pools.

**Results:**

During the study period, the median herd size increased from 74 (2003) to 127 cows (2010), while the prevalence of BVDV infected herds decreased from 0.51 to 0.02 %. The daily milk yield contribution of a single seropositive cow to the entire daily bulk milk was reduced from 1.61 % in 2003 to 0.95 % in 2010 due to the increased herd size. It was observed that antibody levels in bulk milk decreased at national level. Moreover, we found that when testing bulk milk, the SVANOVIR^®^BVDV-Ab can detect a lower prevalence of seropositive lactating cows, compared to the Danish blocking ELISA (0.78 % *vs.* 50 %). Values in the SVANOVIR^®^BVDV-Ab better relate to low concentrations of antibody positive milk (R^2^ = 94-98 %), than values in the blocking ELISA (R^2^ = 23–75 %). For sera, the two ELISAs performed equally well.

**Conclusions:**

The SVANOVIR ELISA is recommended for analysis of bulk milk samples in the current Danish situation, since infected dairy herds e.g. due to import of infected cattle can be detected shortly after BVDV introduction, when only few lactating cows have seroconverted. In sera, the two ELISAs can be used interchangeably.

## Background

Antibody enzyme-linked immunosorbent assays (ELISAs) are commonly used for bulk milk surveillance for bovine viral diarrhoea (BVD). The level of antibodies against BVD virus (BVDV) in bulk milk relates to the prevalence of BVDV seropositive lactating cows in the dairy herd [1]. In Denmark, if the bulk milk is classified as positive, blood is sampled from 25–30 individual animals to find at least one serum-antibody positive animal (with 95 % confidence, assuming a 10 % within-herd prevalence) and to confirm the herd infection status. If no antibody positive animals are detected, the herd is classified as BVD negative, but high bulk milk titers are taken into consideration. If the herd is confirmed positive (i.e. infected) by analysing serum, all animals are sampled and their blood tested for presence of BVDV antibodies. Seronegative cattle are tested for virus to find and cull persistently infected (PI) cattle. Moreover, animal movements are put under restriction until all PI animals have been eliminated from the herd (usually during a one-year-period from first BVDV detection). The Danish blocking ELISA [2, 3] has successfully been used in the national BVD eradication programme [4], which was initiated in 1994 [5, 6]. This study presents data from 2003 to 2010, when Danish dairy herds were screened quarterly by bulk milk testing. During this period the average herd size had increased, which was reflected in an increase in the volumes of milk produced by individual herds. These changes could have resulted in a greater dilution of individual BVDV antibodies in bulk milk.

In Denmark, in 2010, the birth of PI animals was extremely rare, and could be caused by indirect contact to foreign cattle herds or import of pregnant cattle. This is in contrast to the situation in 1994, when herds had seropositive cows. During the eradication programme, the antibody titer in bulk milk in all herds was expected to decrease. An evaluation of the BVD surveillance system is therefore required to ensure that BVDV antibody positive herds are readily detected. The ELISA must be able to detect a low prevalence of antibody positive cows, like a single cow in the population contributing to the bulk milk sample. Early detection of newly infected herds is crucial to control BVD.

The aims of this study were: (i) to investigate how changes in the size of Danish dairy herds and BVD prevalence from 2003 to 2010 might have affected the surveillance based on two antibody ELISAs and (ii) to compare the Danish blocking ELISA [2, 3] and the SVANOVIR^®^BVDV-Ab ELISA (Svanova Boehringer Ingelheim, Uppsala, Sweden) [1, 7–9] for detection of BVDV antibodies in milk and sera.

## Methods

### Population data and BVD status (2003–2010)

Data collected between 2003 and 2010 were obtained from the Danish Cattle Federation. The dataset contained the central husbandry registration (CHR) number of the herds, records of milk production (kg/herd/week), the herd size (number of cows/herd/month) and a quantitative account of the antibody level detected by the Danish blocking ELISA (in blocking percentage) in bulk milk samples. The value of Danish blocking ELISA will from here be referred as bl%. Data on animals being BVDV positive (e.g. date of birth and date of testing) were also included.

To investigate how the herd size and levels in milk production changed during the period, the annual number of dairy herds (from January to December), the herd size, the overall national milk production level and the daily amount of milk (kg) delivered per herd and per cow, were calculated for all years (2003–2010).

The daily milk contribution (in %) of a seropositive lactating cow to the bulk milk, was estimated assuming that (i) all lactating cows had similar milk production and (ii) approximately 17 % (minimum 12 % and maximum 20 %) of the cows present in the herd were dry and did not contribute to the bulk milk. For example, in a herd with 74 cows, we assumed that the average daily number of lactating cows was 62 (minimum 59, maximum 65). Hence, the average individual milk contribution to the bulk milk was 1/62 = 1.61 % (1.54 %; 1.70 %) (Table 1). The proportion of dry cows was based on our knowledge of the Danish dairy industry.

**Table 1:** Changes in number of milking cows per Danish herd and their individual contribution to the bulk milk

Parameter		2003			2010	
	80 %	83 %	88 %	80 %	83 %	88 %
a	59 (463)	62 (480)	65 (509)	101 (948)	105 (984)	111 (1043)
b	25 (28)	25 (27)	23 (26)	29 (32)	28 (31)	26 (29)
c	1.70 (0.22)	1.61 (0.21)	1.54 (0.20)	0.99 (0.11)	0.95 (0.10)	0.90 (0.10)

Parameters a, b and c were estimated based on data from the Danish diary industry, assuming that usually 17 % of the cows in a herd are dry and not contributing to the bulk milk. Therefore, usually 83 % of the cows are lactating, 80 % is the minimum and 88 % is the maximum proportion of lactating cows. a) Number of lactating cows/herd/day according to median herd size in 2003 (74 cows) and 2010 (127 cows), b) amount of milk produced (kg) per cow/day and c) daily contribution of a single cow to the bulk milk (in %). The estimates for the largest herds in 2003 (579 cows) and 2010 (1185 cows) are shown in brackets

To study changes in the infection status, the prevalence of herds with viremic animals was estimated by calculating the annual proportion of herds with at least one BVDV positive animal. Herds that ceased with production during the year were also considered.

The Danish bulk milk values (in bl%) were investigated for each year, using data on antibody detection in bulk milk.

The freeware R (version 2.13.2, R core development team, 2010) and Excel (Microsoft Office, 2007) were used for data analysis.

### Antibody ELISAs

The Danish blocking ELISA was performed as previously described [2]. For this test, the sensitivity (Se) and specificity (Sp) when applied to individual milk samples have not been estimated. In bulk milk, when the prevalence of infected herds was 26 %, estimates of Se and Sp were 100 % and 62 % respectively, using a cut-off bl% of 50 [10].

Currently, the decision criteria used by the Danish Cattle Federation to consider a herd as positive, based on bulk milk testing, is an increase in the blocking percentage to 50 % [3, 5, 10] and/or two consecutive bulk milk samples ≥ 20 %. In this study, individual milk, bulk milk samples and milk pools were defined as positive if the bl% was above 0, according to the current antibody levels in the national dairy population. In most dairy herds the bl% was close to 0 in 2010 (see results). This could be due to eradication of BVD from Denmark, and/or because the dilution of individual BVDV antibodies in bulk milk is too high to allow detection by the used ELISA.

In individual serum, the Se and Sp are 96.5 % and 97.5 % respectively, if a cut-off bl% of 50 is used [2].

The ELISA SVANOVIR^®^BVDV-Ab [1, 7–9] was performed according to the instructions from the manufacturer. Values were calculated as percentage positivity (PP), and individual milk samples were considered positive if PP ≥ 9. In this study, diluted milk samples and artificial pools of milk representing bulk milk were classified as positive if PP was ≥ 2. According to the manufacturer, this value corresponds to a low antibody level in the herd. In serum, the reported Se and Sp are 100 % and 98.2 % respectively [11], using PP of 15 as indicative of an antibody positive sample.

### Milk and serum testing

Individual milk and serum samples were obtained from three Danish dairy herds (A, B and C), which contained BVD antibody positive and antibody negative lactating cows. Herd A was determined to be a BVDV positive herd (5^th^ October 2011) due to an increase in the bulk milk antibody titre after birth of PI calves. Herd B was suspected of BVDV infection following an increase in BVDV antibody titre in bulk milk in November 2010. A serological analysis of the herd revealed that imported pregnant heifers had given birth to PI animals in January 2010. The route of BVDV introduction in herd C is unknown. During the study period (2010–2011), the herd size in herds A, B and C was around 350, 180, and 259 cows, respectively.

To evaluate the impact of larger herds and of a reduced in-herd prevalence of BVDV antibody positive cows on the surveillance system for Danish dairy herds, the Danish blocking ELISA and the SVANOVIR were compared. The minimum prevalence of BVDV antibody positive cows needed to be able to detect a positive antibody titre in a bulk milk sample was compared. Experiments were carried out by a) analysing dilutions of antibody positive individual milk samples and b) analysing artificially made bulk milk samples with a known proportion of antibody positive milk.

Dilution experiments were performed on individual samples from herd A, where serum and milk were collected from 303 lactating cows. Of these, 149 cows were selected randomly for our study. Thereafter, milk samples from 77 cows that were positive in both ELISAs in milk and serum were divided into three groups according to their antibody titer in the Danish blocking ELISA: low (L, n = 19 cows), medium (M, n = 38) and highly (H, n = 20) positive, according to the 1^st^ and 3^rd^ quartiles of the bl% in milk (12 % and 34 %, respectively). The minimum and maximum bl% were 0.3 % and 96 %. Thereafter, three cows in group L, three cows in group M and four cows in group H were randomly selected. Milk and serum samples from these ten lactating cows were serially two-fold diluted in seven dilution steps from 1/2 up to 1/128 in BVDV antibody negative milk or serum, respectively. BVDV antibody negative milk and serum samples used for the dilutions were tested negative in both ELISAs.

Artificial bulk milk samples were made from ten positive and 31 negative cows from herds B and C. In this experiment, cows were classified as positive or negative according to bl% in milk. Antibody positive and negative milk pools were made; cows contributing to the positive milk pool had milk bl% between 89 % and 97 %, and serum bl% between 98 % and 99 %. Nineteen artificial bulk milk samples of 5 ml. each were constructed, they contained 10–100 % BVDV antibody positive milk, diluted in antibody negative milk. To focus on bulk milk series with low antibody levels, we additionally analysed 12 artificial bulk milk samples with concentrations of positive milk from 2.5 to 30 %, with increments of 2.5 percentage points.

Original bulk milk samples, collected from herds B and C, were analysed using both ELISAs to compare the antibody levels after removal of PI animals.

### Linear regression model for artificial pools of milk

For the artificial pools of milk, a simple linear regression model was used to examine the association between the concentration of positive milk in an artificial bulk milk sample as a dependent variable and the level of antibodies measured by each ELISA (as an explanatory variable). A log transformation was used for both variables. The coefficient of determination (R^2^) was calculated to estimate the variation in the proportion of positive milk in a pooled sample, which can be explained by the values obtained with the ELISA used.

## Results

### Descriptive statistics on herd size and BVD status in Denmark

**Fig. 1:**
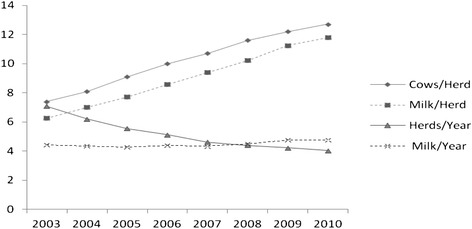
Changes in the size and milk production of Danish dairy herds from 2003 to 2010. Cows/Herd = median herd size (divided by 10); Milk/Herd = milk produced per herd (in 100,000 kg). Herds/Year = number of Danish dairy herds (in 1,000), which delivered milk from January to December; Milk/Year = national milk production (in billion kg of milk)

Between 2003 and 2010, the number of dairy herds delivering milk during a full year period decreased from 7075 to 4037. The median herd size was 74 cows in 2003 and 127 cows in 2010 (Fig. 1). The two largest herds had 579 and 1185 cows, respectively. The overall national milk production remained at the same level, with approximately 4.4 billion kg in 2003 and 4.7 billion kg in 2010 (Fig. 1).

The milk yield per cow increased by 3–4 kg during the period, but due to the increased herd size, the contribution of a single animal to the daily herd’s production decreased from 1.61 to 0.95 % when comparing herds of median size, and from 0.21 to 0.10 % when comparing the largest herds present in 2003 and 2010 (Table 1).

In 2003, the prevalence of herds with at least one BVDV positive animal was 0.51 % (39/7731), whereas in 2010 only 0.02 % (1/4255) were found to be virus positive. This was due to import of pregnant cows carrying PI calves (herd B), which were not tested for BVD virus when born.

In 2003, a total of 31,345 bulk milk samples were analysed. Of those, 95 % had a bl% below the cut-off 50 %. In 2010, 17,298 bulk milk samples were tested, 75 % of which had a bl% of 0 (below detection level), while the remaining 25 % had a median bl% of 5 (3^rd^ quartile = 9 %). The maximum value obtained was bl% = 80 in herd B.

### Seroprevalence in herd A

In herd A, the prevalence of individual antibody positive milk and serum samples detected by the blocking ELISA was 56 % and 71 %, respectively. Five cows tested positive in milk but not in serum. The SVANOVIR tested 87 % and 69 % positive, respectively, since 27 cows tested positive in milk but not in serum. These 27 cows were not considered for the dilution experiments, as they were not confirmed as true positives. Only samples from animals positive in both tests, in both milk and sera were used.

### Dilution series in milk and serum

**Fig. 2:**
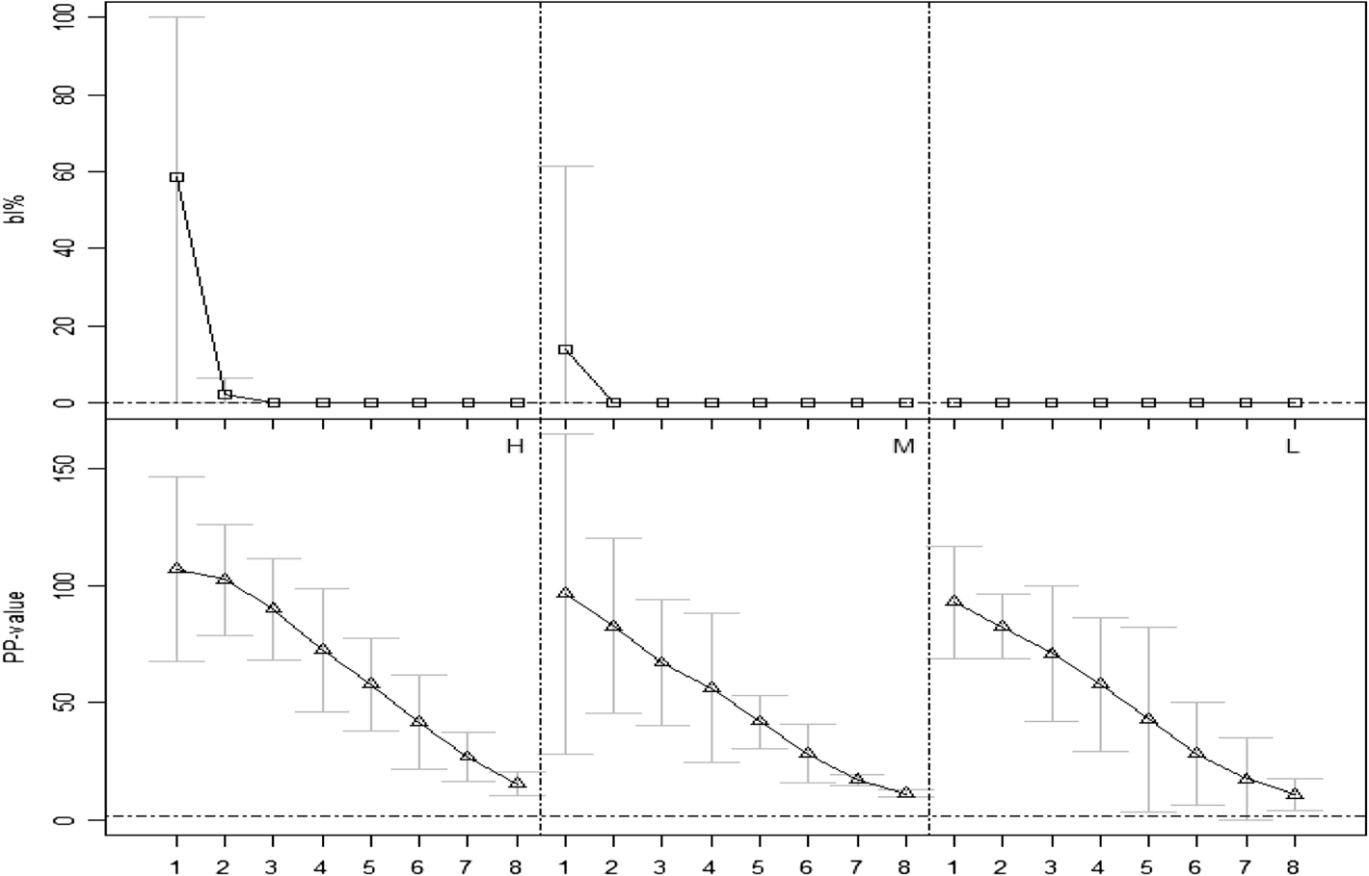
Results obtained on diluted individual milk samples. On the x-axis, 1 corresponds to the undiluted sample, while 2-8 represent dilution steps 1/2 up to 1/128. □ = mean bl% using the Danish blocking ELISA; ∆ = mean PP-value using the SVANOVIR. Grey bars represent 95 % confidence interval around each mean, H: highly positive group (n = 4), M: medium positive group (n = 3), and L: low positive group (n = 3). Horizontal dashed lines represent the cut-offs (bl% = 0 and PP = 2), above which samples were classified as antibody positive. N.B. Test values below zero do not have any biological meaning, and thus, in the 95 % confidence intervals we set the minimum bar limit to 0. We proceeded in a similar way for the maximum limit of the blocking ELISA, which cannot have values > 100 %

In the milk dilution experiments, the SVANOVIR was positive in all ten milk samples in all dilution steps to 1/128 (Fig. 2). When using the blocking ELISA two cows from the highly positive group were positive in milk in the blocking ELISA at dilution 1/2, while all ten animals were negative at dilutions ≥ 1/4. Moreover, animals in the low positive group (L) showed bl% = 0 even in the undiluted sample (Fig. 2).

**Fig. 3:**
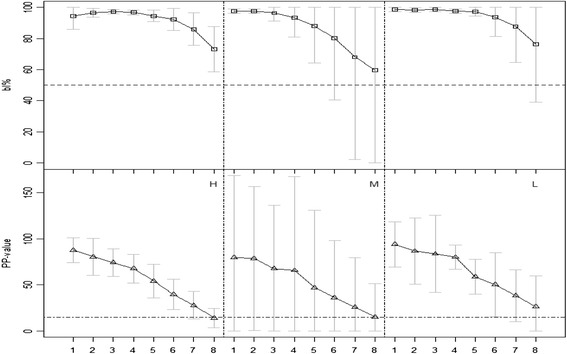
Results obtained on diluted individual serum samples. On the x-axis, 1 corresponds to the undiluted sample, while 2-8 represent dilution steps 1/2 up to 1/128. □ = mean bl% with the Danish blocking ELISA; ∆ = mean PP-value using the SVANOVIR. Grey bars represent 95 % confidence interval around each mean, H: highly positive group (n = 4), M: medium positive group (n = 3), and L: low positive group (n = 3). Horizontal dashed lines represent the cut-offs (bl% = 50 and PP = 15) above which samples were classified as antibody positive. N.B. Test values below zero do not have any biological meaning, and thus, in the 95 % confidence intervals we set the minimum bar limit to 0. We proceeded in a similar way for the maximum limit of the blocking ELISA, which cannot have values > 100 %

In the serum dilution experiment the two ELISAs had comparable results (Fig. 3). One cow from group M was negative in the blocking ELISA at dilution 1/64 (bl% = 45), the same cow was antibody negative at the same dilution in the SVANOVIR (PP = 14), together with another cow from the same group (PP = 13).

### Artificial bulk milk series

In artificial bulk milk series with 10–100 % BVDV antibody positive milk, the relation between test values and the concentration of positive milk was significant for both tests (*P*-value < 0.0001). The R^2^ was 75 % for the blocking ELISA and 98 % for the SVANOVIR. However when analysing pools with low antibody titters, 2.5–30 % positive milk, no significant relation between the bl% and the concentration of positive milk (*P*-value = 0.12) was obtained. The R^2^ was 23 % for the blocking ELISA and 94 % for the SVANOVIR.

### Bulk milk from herds B and C

**Fig. 4:**
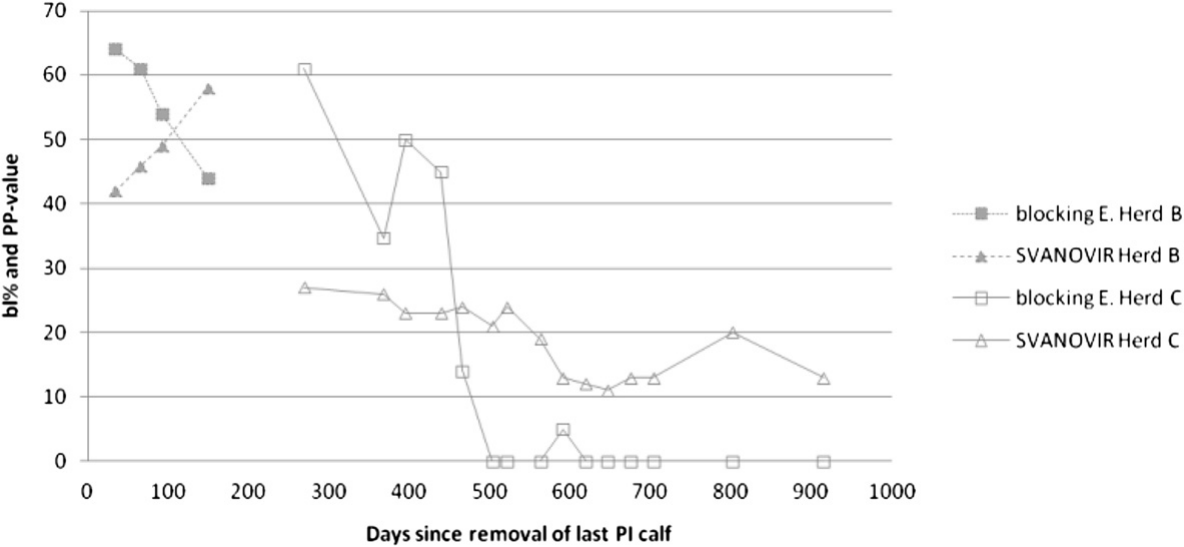
Change in bulk milk values after removal of all PI calves (herds B and C). Results from two dairy herds during a 149 days evaluation for herd B and 915 days for herd C. The y-axis represents the bl% and PP-values according to the blocking ELISA and the SVANOVIR respectively, while the x-axis represents the number of days since removal of the last born PI calf and the bulk milk sampling. Four and fifteen samples were tested from herds B and C respectively

Bulk milk samples from the field were analysed and both ELISAs classified herd B as positive (bl% = 44; PP = 58) 149 days after removal of the last born PI calf. Herd C was classified negative in the blocking ELISA after 503 days (bl% = 0), but still remained positive in the SVANOVIR after 915 days (PP = 13) (Fig. 4).

## Discussion

The Danish eradication programme was initiated in 1994 when 39 % of the herds were expected to contain PI animals and the average herd size was 42 cows [5]. Since then the herd structure has changed and the BVD incidence has decreased. The results from the present study should provide important information on how to proceed in the Danish BVD surveillance system.

Although important changes occurred in the number and size of milking herds, the Danish milk production remained at the same level between 2003 and 2010 (Fig. 1). A slight increase in milk production per cow was observed, but the dilution of individual antibodies increased steadily (Table 1) due to increased herd size. The increase in the median Danish herd size was quite sudden (Fig. 1) and the contribution of a single antibody positive animal in a herd has become more difficult to detect in bulk milk testing (Table 1).

Our epidemiological investigations showed that BVD is at present an exotic disease in Denmark, because the prevalence of herds with viremic cattle decreased steadily during the investigated years, now only sporadic cases are detected.

The antibody titer in Danish bulk milk samples decreased and most of the samples were below the detection level of antibodies in 2010. Accordingly, we used cut-off bl% = 0 to classify a milk sample as positive with the blocking ELISA. Using this criterion, most Danish dairy herds were considered to be naïve to BVDV. Therefore, we did not have access to bulk milk samples and individual milk/serum samples from more than the three previously infected herds (A, B, and C), for our experiments. We used the dilution experiments and artificial pools of milk, to investigate the impact of a changed herd structure and antibody dilution level, comparing the original Danish blocking ELISA and the new SVANOVIR ELISA. Moreover, to represent the bulk milk in our experiments, we assumed that all milking animals produced a similar amount of milk and that all seropositive animals had the same antibody titre. In reality this is not the case, and thus, we used a simplification. For instance, the concentration of antibodies in individual milk could be higher at the beginning and at the end of lactation [8] or if a cow carries a PI calf [12]. Nevertheless, we think that our experiments give important information on the comparison of the two tests when used for bulk milk testing. As shown by Graat *et al*. [13], for infectious bovine rhinotracheitis (IBR), the threshold prevalence of antibody positive cows at which the ELISA used can classify the bulk milk as positive, is a parameter that needs severe consideration, since it affects the detection time and the performance of the surveillance system.

We found that the SVANOVIR ELISA can classify the bulk milk as positive, at a lower prevalence of seropositive cows (and thus sooner after BVDV introduction) than the blocking ELISA. Therefore, the SVANOVIR ELISA could be used to optimize the Danish surveillance system in dairy herds.

In the dilution experiments, it was found that the SVANOVIR could detect one single antibody positive animal, corresponding to an individual contribution of 0.78 % (1/128) to the bulk milk (Fig. 2) in more than half of the herds (with ≤ 128 milking cows). We considered the seven dilution steps sufficient, since a herd with 128 lactating cows would have approximately 145 cows (considering that at least 12 % are dry), which is close to the most common herd size in Denmark.

On average, one cow can contribute to 0.90–0.99 % of the overall herd’s production (Table 1), and therefore recently infected herds could be detected soon after infection when the number of antibody positive animals is low. In the largest herds, with 129–1043 lactating cows and a herd size of 147 and 1185 cows respectively, two to nine animals should be antibody positive to result in a positive BVD bulk milk antibody value. With the blocking ELISA, at least 50 % of the milking cows should be positive (Fig. 2) to be detectable in a bulk milk sample. This finding is in agreement with the prevalence of antibody positive milking cows in herd A at the time of BVD detection by bulk milk testing.

Regarding the animals in the “L” group, which showed milk bl% = 0 in all dilution steps (Fig. 2) it must be mentioned that, any ELISA when repeated, is very unlikely to give exactly the same values. In the first test (when we tested all cows for the first time), the three animals of the “L” group had bl% > 0 in milk. Those animals were also positive in milk in the SVANOVIR and in serum with both ELISAs and were assumed to be true positives. Nevertheless, when we repeated the test for the dilution trials, these cows were negative in the undiluted milk sample with the blocking ELISA (Fig. 2). This means that especially in low positive animals, the repeatability and robustness on milk samples is low for the blocking ELISA.

Furthermore, the linear regression model with the SVANOVIR’s values as x-variable explained 94–98 % of the variation in the concentration of positive milk pool used (y-variable), while the model with the blocking ELISA could explain 23–75 %. At low concentrations of positive milk (2.5–30 %) the explanatory power of both tests was lower and was not significant for the blocking ELISA. Thus, especially when analysing bulk milk from herds with few positive cows, the SVANOVIR relates better to the low prevalence of positive lactating cows contributing to the bulk milk.

A low Se in milk is a common problem for blocking ELISAs and a high prevalence of seropositive lactating cows could be needed before bulk milk samples are antibody positive [14]. Therefore, the threshold prevalence (50 %) estimated in the dilution experiments to have positive bulk milk appears correct. In this kind of ELISA system, the cause of low Se in milk and high Se in paired serum could be explained by a well-buffered environment in serum, whereas milk samples may have a low pH due to acidic bacteria that make the milk sour. This creates a suboptimal environment for the antigen-antibody binding. Moreover, antibody levels are lower in milk than in serum [15].

With the SVANOVIR, in herd A, 27 cows were positive in milk but negative in serum. This was a surprising finding, because it was expected that milk would have a lower titre than serum [15]. According to the veterinarians who carried out the sampling, mismatching of milk and serum samples was unlikely, especially since a very high percentage of animals (27/149 = 18 %) showed this unexpected result. With the Danish blocking ELISA, this percentage was by far lower (5/149 = 3.4 %). Similar results to ours were found in the study by Niskanen *et al*. [8]. Thus, higher positivity in milk compared to serum can sometimes be found, when the SVANOVIR is used. With this test, serum is tested after dilution, while milk is tested undiluted. Schrijver and Kramps [16] suggested that when indirect ELISAs are used, as is the case with the SVANOVIR, samples should be diluted before analysis to avoid unspecific binding of antibodies. Unspecific binding is a common problem for indirect ELISAs, e.g. because non-specific antibodies bind to the well and, depending on the washing conditions, they will be detected by the conjugated antibody.

If the SVANOVIR is selected for bulk milk testing, a preliminary screening of all dairy herds present in the country could be made, to define the baseline national antibody status (according to the new test). In herds with positive or doubtful bulk milk reactions a pooled milk sample from cows in their first lactation, which should be naive to BVDV, could be tested. Using this system, new infections are detected and the proportion of false positive bulk milk samples could be defined for the SVANOVIR ELISA. This is necessary, since the investigation of a herd suspected of having BVDV based on antibodies in bulk milk, will require additional testing of individual animals at the cost of about 800–900 Euro.

Regarding results from bulk milk samples of herds B and C (Fig. 4), we showed that the SVANOVIR required more time to become negative again after removal of PI animals. At this stage, alternative testing strategies are used in the herd, like testing of serum from young animals born after culling of PIs from the herd [17]. Moreover, while in herd C both tests showed decreasing bulk milk antibody titers; in herd B, the SVANOVIR showed an increasing trend after removal of the last born PI, while the Danish blocking ELISA had steadily decreasing values (Fig. 4). These differences between tests could be caused by more PI(s) cattle being born in the herd but having died before being detected by the veterinarians, who carried out the sampling according to the BVDV programme. The introduction of such a PI calf could have been signalled in the bulk milk by the SVANOVIR and not by the blocking ELISA.

Finally, when analysing serum samples for antibodies to BVDV, we found that both ELISA’s has similar sensitivity (Fig. 3). Both ELISA’s can be used to analyse serum from newborn calves, imported cattle (e.g. pregnant cows which could carry PI calves) or to conduct follow-up studies in dairy herds suspected of being infected.

## Conclusions

The combination of increased dilution of individual antibodies in bulk milk and decreased BVDV antibody prevalence is a challenge for the surveillance programmes. In countries with large dairy herds and with low BVDV prevalence (e.g. Denmark), the SVANOVIR could be preferred for an early warning surveillance system based on bulk milk testing, because a lower prevalence of seropositive milking cows can be detected (compared to the situation where the Danish blocking ELISA is used). Analysis of individual blood could be performed using either of the two ELISAs.
